# The Role of the Hfq Protein in Bacterial Resistance to Antibiotics: A Narrative Review

**DOI:** 10.3390/microorganisms13020364

**Published:** 2025-02-07

**Authors:** Sylwia Bloch, Grzegorz Węgrzyn, Véronique Arluison

**Affiliations:** 1 Department of Molecular Biology, University of Gdansk, Wita Stwosza 59, 80-308 Gdansk, Poland; sylwia.bloch@ug.edu.pl; 2 Laboratoire Léon Brillouin, UMR 12 CEA/CNRS, Bâtiment 563, Site de Saclay, 91191 Gif-sur-Yvette, France; 3 UFR Science Du Vivant, Université Paris Cité, 35 Rue Hélène Brion, 75013 Paris, France

**Keywords:** Hfq protein, antibiotic resistance, gene expression regulation, influx and efflux pumps, outer membrane, porins, biological membranes, plasmids, transposons

## Abstract

The antibiotic resistance of pathogenic microorganisms is currently one of most major medical problems, causing a few million deaths every year worldwide due to untreatable bacterial infections. Unfortunately, the prognosis is even worse, as over 8 million deaths associated with antibiotic resistance are expected to occur in 2050 if no new effective antibacterial treatments are discovered. The Hfq protein has been discovered as a bacterial RNA chaperone. However, subsequent studies have indicated that this small protein (composed of 102 amino acid residues in *Escherichia coli*) has more activities, including binding to DNA and influencing its compaction, interaction with biological membranes, formation of amyloid-like structures, and others. Although Hfq is known to participate in many cellular processes, perhaps surprisingly, only reports from recent years have demonstrated its role in bacterial antibiotic resistance. The aim of this narrative review is to discuss how can Hfq affects antibiotic resistance in bacteria and propose how this knowledge may facilitate developing new therapeutic strategies against pathogenic bacteria. We indicate that the mechanisms by which the Hfq protein modulates the response of bacterial cells to antibiotics are quite different, from the regulation of the expression of genes coding for proteins directly involved in antibiotic transportation or action, through direct effects on membranes, to controlling the replication or transposition of mobile genetic elements bearing antibiotic resistance genes. Therefore, we suggest that Hfq could be considered a potential target for novel antimicrobial compounds. We also discuss difficulties in developing such drugs, but since Hfq appears to be a promising target for drugs that may enhance the efficacy of antibiotics, we propose that works on such potential therapeutics are encouraged.

## 1. Introduction

Despite the discovery of antibiotics a century ago and the subsequent development of antibiotic therapies, bacterial infections remain a significant medical challenge. In 2019, there were 13.7 million deaths worldwide linked to various infections, with 7.7 million of these attributed to bacterial pathogens, either resistant or susceptible to antimicrobial drugs [1]. Over 50% of these fatal cases were caused by bacteria from five species: *Escherichia coli*, *Klebsiella pneumoniae*, *Pseudomonas aeruginosa*, *Staphylococcus aureus*, and *Streptococcus pneumoniae*. The rapid development of antibiotic resistance poses an even greater threat. In 2021, approximately 1.2 million deaths were directly attributable to antimicrobial resistance, while 4.7 million deaths were associated with it [2]. Forecasts based on current trends and predictions suggest that annual deaths due to antibiotic resistance could reach 1.9 million by 2050, with 8.2 million deaths linked to infections caused by resistant bacteria. If no novel antimicrobial drugs are developed, between 2025 and 2050, an estimated 39 million deaths will be directly attributable to antibiotic resistance, and 169 million deaths will be associated with infections involving resistant bacteria [2].

These findings underscore the emerging antibiotic resistance crisis, driven by the selective pressure on bacterial strains to evolve resistance to antimicrobial agents, resulting in infections that are either difficult or impossible to treat [3]. Consequently, various health authorities have emphasized the urgent need for the discovery of new, effective antimicrobial agents and/or the development of novel strategies to combat bacterial infections to avoid potentially catastrophic, life-threatening pandemics in the near future [4].

This is particularly critical as some bacteria have developed resistance to multiple classes of antibiotics, making them a significant public health problem. These multidrug-resistant (MDR) bacteria, often linked to hospital-acquired/nosocomial infections, are of particular concern. Notable examples of MDR bacteria include Methicillin-resistant *S. aureus* (MRSA); carbapenem-resistant Enterobacteriaceae, such as *Klebsiella* and *E. coli* strains; MDR *Mycobacterium tuberculosis*; and extended-spectrum β-lactamase (ESBL)-producing Gram-negative bacteria [1].

In light of this, the search for new antibacterial compounds is not only highly encouraged but is becoming an absolute necessity. Various innovative approaches are being explored, including investigations into plant-derived products [5], the discovery of novel compounds from hard-to-reach environments [6], the use of bacteriophages (viruses that infect bacterial cells) in “phage therapy” [7], and the application of phage-encoded enzymes to destroy bacterial cell membranes and walls as antibacterial agents [8], among others.

In addition to identifying previously unknown compounds with antibacterial properties and developing strategies for utilizing naturally occurring agents like bacteriophages, another promising approach for introducing novel antimicrobial therapies is the discovery of specific therapeutic targets. Uncovering new molecular mechanisms of action for particular proteins [9] and/or the identification of specific processes occurring in bacterial cells [10] represents a key avenue for drug development. One advantage of these strategies is the ability to leverage modern techniques, such as molecular modeling, to design new therapeutic molecules or enhance the efficacy of existing antibiotics by disrupting resistance mechanisms. Therefore, identifying new targets for antimicrobial resistance offers a particularly promising path for the development of novel antibacterial drugs.

In this light, we aimed to provide an overview and discuss properties of the bacterial Hfq protein linked to the sensitivity/resistance of microorganisms to antibiotics. Gaining knowledge in this field may facilitate searching for novel drugs that could enhance the susceptibility of bacteria to antimicrobial agents, thus being potential drugs in therapies against pathogenic bacteria that reveal different levels of antibiotic resistance. In fact, studies on the effects of Hfq on antibiotic resistance are quite novel; thus, we supposed they deserve such a review article.

**Figure 1:**
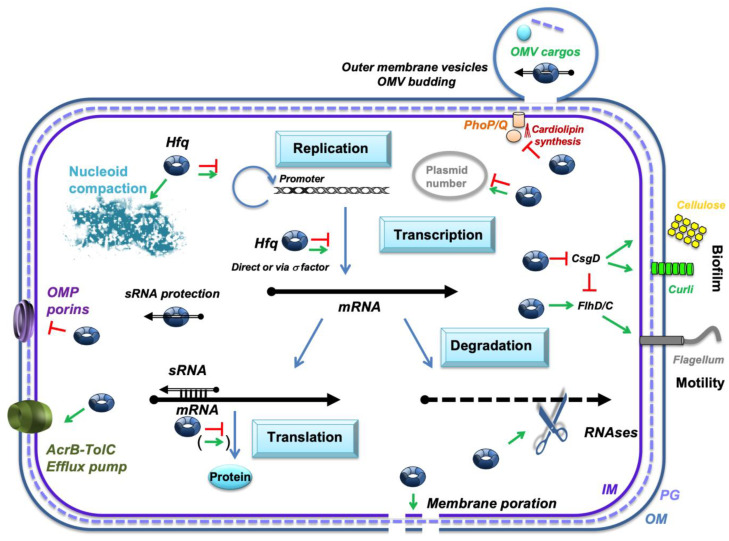
Network of Hfq-dependent processes and regulations. Hfq plays a crucial role in the post-transcriptional regulation of gene expression, including genes involved in antibiotic resistance. It primarily exerts its effects through interactions with small noncoding RNAs (sRNAs). One key mechanism is the regulation of efflux pumps, such as AcrB, which actively expel antibiotics from bacterial cells. In addition, Hfq modulates antibiotic influx by regulating outer membrane proteins (Omps). Beyond its role in antibiotic resistance, Hfq is involved in regulating processes like flagellum formation and biofilm matrix production, both of which enhance bacterial survival and resistance to antibiotics. Hfq also influences the expression of plasmid-encoded enzymes, such as β-lactamases, which modify or degrade antibiotics. Furthermore, Hfq participates in broader regulatory networks that include responses to environmental signals, sigma factor production, nucleoid compaction, a membrane’s lipid composition, and outer membrane vesicle (OMV) budding. These diverse functions underscore Hfq’s central role in bacterial adaptation and survival under stress conditions. The sRNA regulators controlling mRNAs are shown as black arrows; Hfq is represented by a blue toroidal hexamer; mRNAs are depicted as thick black lines; the 5′ and 3′ ends of the mRNA are depicted by a “ball and arrowhead”, respectively; positive and negative regulations are indicated by red arrows and green horizontal bars, respectively; and the dotted line symbolizes peptidoglycan (PG) between outer (OM) and inner (IM) membranes.

The Hfq protein is widely recognized as an RNA chaperone that facilitates interactions between different RNA species, [11]. Initially, Hfq was discovered as a factor required for the propagation of *E. coli*-infecting bacteriophage Qβ, specifically for the synthesis of the RNA minus-strand, complementary to the phage RNA genome [12]. However, subsequent studies have shown that Hfq is particularly important for the annealing of small noncoding regulatory RNAs (sRNAs) with their mRNA target. Later, Hfq was also found to be involved in a wide range of other cellular processes, including ribosome biogenesis, DNA compaction, and the establishment of various protein–protein interactions among others [13,14,15] (Figure 1). Indeed, a broad range of pleiotropic phenotypes resulting from Hfq deletion have been observed in *Escherichia coli* [16]. The RNA annealing activity of Hfq is mainly due to its N-terminal region (approximately 65 residues), which adopts a characteristic fold, called the Sm-fold. This region forms a homohexameric ring structure [17,18]. In some bacteria, like *E. coli*, Hfq additionally has a C-terminal region of about 35 residues. This region extends outward from the N-terminal torus [19]. Initially thought to be intrinsically disordered [18], recent evidence shows that the C-terminal region of *E. coli* Hfq can form a β-rich, amyloid-like structure [20,21]. While the exact role of this C-terminal region in RNA annealing is debated, it seems dispensable for most sRNA-mediated regulatory processes [19,22]. However, its absence may affect some specific sRNA regulatory pathways [23,24,25,26].

## 2. Methods

This is a narrative review, based on information available in the scientific literature. The PubMed database (https://pubmed.ncbi.nlm.nih.gov, accessed on 1 February 2025) was searched using the terms “Hfq” and “antibiotic resistance”. Fifty-three records were identified by such a search. Non-English articles were excluded from further analysis, as were papers describing studies in which antibiotics were used only as tools for investigations (for example, the use of plasmids carrying antibiotic resistance genes solely as vectors for genes introduced into *hfq* mutant strains). After excluding such articles, 37 articles were considered relevant and taken into account for detailed analysis. Other articles cited in this review are papers describing the antibiotic resistance phenomenon, properties of the Hfq protein, and other issues related to the subject of this work.

## 3. Evaluating Hfq Influence on Antibiotic Resistance

Several methods are available to evaluate antibiotic resistance in the absence of Hfq, ranging from traditional culturing techniques to advanced molecular and imaging assays. The most commonly used methods include the following: (i) disk diffusion (the Kirby–Bauer method), where an antibiotic-impregnated paper disk is placed on an agar plate and the zone of inhibition measured to determine resistance [27,28]; (ii) the minimum inhibitory concentration (MIC) measurement, which determines the lowest concentration of an antibiotic that inhibits bacterial growth [29,30]; (iii) fluorescent live/dead imaging, where a combination of two nucleic acid-binding stains is used: one stain, such as SYTO 9, penetrates all bacterial membranes and stains the cells (in green in the case of SYTO 9), while the other stain such as propidium iodide (PI) only penetrates cells with damaged membranes, producing red fluorescent cells in the case of PI [31,32]; (iv) whole genome sequencing, which provides detailed information about all mutations in resistance genes [33,34]; (v) RNAseq, used to analyze the whole transcriptome of a resistant bacterium [35,36]; (vi) antibiotic resistance phenotypic profiling, which tests bacterial isolates against a panel of antibiotics [37,38]; and (vii) in vivo antibiotic accumulation or susceptibility, which evaluates bacterial resistance within a biological system, such as in animal models or an organoid [39].

In parallel, quantitative analyses of antibiotic accumulation within a population of bacteria can also be conducted. To quantify the intra-bacterial antibiotic concentration, a fluorescent antibiotic can be used. For instance, ciprofloxacin naturally fluoresces and a measurement of its accumulation in the cell can be performed in bulk or at the single-cell level [40,41,42]. Unlike bulk measurements, the single-cell approach allows for monitoring the accumulation dynamics in intact, live cells over time, without the need for bacterial population synchronization. It also enables the tracking of accumulation kinetics in the same cell throughout the course of the experiment. This approach is important as cell-to-cell variation in antibiotic accumulation is observed [43].

## 4. The Involvement of the Hfq Protein in the Regulation of Bacterial Resistance to Antibiotics

### 4.1. Early Studies and Effects of Hfq on Biofilm Formation

The first reports indicating that Hfq may be involved in antibiotic resistance in *E. coli* were published less than 20 years ago. It was found that this protein can regulate the expression of genes coding for some efflux pumps that are able to remove antibiotics from cells, like AcrAB, EmrE, MdfA, and especially MdtEF [44]. Initially, it was thought that Hfq might support antibiotic resistance only indirectly, for example, by enhancing the ability to form biofilms, as biofilms build a barrier for antibiotics.

Indeed, *hfq* mutants or cell with an impaired expression of this gene were found less prone to form biofilms. This was observed in uropathogenic *E. coli* [45], *P. aeruginosa* [46], *Pseudomonas fluorescens* [47], and *Shigella sonnei* [36], and this was correlated to increased sensitivity to antibiotics. In *P. aeruginosa*, a small RNA molecule PqsS was identified, which interacts with Hfq and is involved in the regulation of both biofilm formation and antibiotic resistance [32]. This discovery may show a way by which Hfq controls the ability of bacteria to produce biofilm, thus modulating antibiotic accessibility to cells.

### 4.2. Hfq-Mediated Regulation of Efflux Pumps, Porins, and Lipopolysaccharide Composition

Transcriptomic analysis performed with an *hfq* deletion mutant of *Yersinia pestis* indicated that a lack of the Hfq protein resulted in an alteration of levels of over 200 transcripts, including those whose products are involve in the processes of virulence and resistance [48]. In fact, testing *hfq* mutants of *E. coli* revealed that they are more susceptible to different antibacterial agents than wild-type cells, including some antibiotics, like cefamandole, chloramphenicol, nalidixic acid, novobiocin, oxacillin, and rhodamine 6G [49]. Among the antibiotic resistance mechanisms, one key parameter is the antibiotic efflux, which reduces intracellular antibiotic concentrations. This mechanism arises from the activity of transmembrane efflux pumps that recognize and expel a wide range of antibiotics [50,51]. The resistance nodulation division (RND) efflux pump family is the most well characterized [52]. Specifically, the major *E. coli* RND efflux pump AcrAB-TolC plays a crucial role in the development of multidrug-resistant phenotypes [53]. This pump is composed of three proteins: AcrB, an active drug transporter that utilizes the proton motive force across the inner membrane; TolC, which forms an outer membrane (OM) channel; and AcrA, a periplasmic linker [54,55,56]. The AcrA/AcrB/TolC polypeptides function together, and if any one of the subunits is absent or inactive, the entire pump’s activity is compromised. Previous studies have demonstrated that Hfq is involved in regulating *acrB* expression [49,57]. Since the deletion of the *acrB* gene, coding for the AcrB subunit of the efflux pump, decreased the effect of Hfq deficiency on antibiotic resistance, it was concluded that the Hfq protein enhances antibiotic resistance by the positive post-transcriptional regulation of *acrAB* expression [49]. Similar results were reported recently in *Aeromonas veronii*, where the deletion of *hfq* resulted in increased sensitivity to trimethoprim, while the ectopic overexpression of *acrA* and *acrB* genes recovered trimethoprim resistance in the Δ*hfq* mutant [57]. Recently, experiments conducted with *E. coli* strains devoid of the *hfq* gene or its part encoding the C-terminal domain of Hfq demonstrated a more effective accumulation of ciprofloxacin in mutant cells (relative to controls), irrespective of the presence of the active AcrAB-TolC efflux pump [41].

On the other hand, outer membrane porins (Omps) play a crucial role in regulating cellular permeability to antibiotics [58,59]. OmpC and OmpF are essential Omps in *E. coli* that facilitate the passive diffusion of small hydrophilic molecules, including sugars, aminoacids or certain antibiotics. Structurally, both OmpC and OmpF are β-barrel-shaped proteins that form channels in the OM [60,61]. While they share a similar structure, they differ in their pore size and permeability [62,63,64]. OmpC forms a narrow channel, whereas OmpF forms a wider channel that permits the diffusion of larger molecules [64,65]. The reduced expression or loss of porins is a common mechanism through which bacteria acquire resistance. Mutations in the *ompC*/F genes can lead to a reduction in channel size or a complete loss of porin function. These mutations are frequently observed in multidrug-resistant *E. coli* clinical isolates [66]. Hfq is known to modulate the expression of mRNAs encoding various outer membrane proteins regulated by noncoding RNAs, including several genes that code for porins [67,68,69]. The overexpression of the gene encoding a small regulatory RNA, MicF, that, in an Hfq-mediated regulatory process, decreases the efficiency of *ompF* expression (thus resulting in a lower abundance of the OmpF porin), caused a reduced accumulation of ciprofloxacin [41]. Nevertheless, recent results indicate that Hfq can be involved in antibiotic resistance processes independently of the functions of porins. Indeed, Hfq influences ciprofloxacin accumulation in *E. coli* independently of *ompC* and *ompF* post-transcriptional regulation [42]. These findings supported the hypothesis that Hfq modulates antibiotic transmembrane influx and efflux through sRNA-independent mechanisms. Accordingly, Hfq could influence both protein- and lipid-mediated pathways for fluoroquinolone (FQ) entry into the cell. One possibility is that Hfq forms pores that directly allow FQ influx [70,71]. Hfq may enhance lipid-mediated FQ transport by influencing membrane integrity [42] and also by regulating the pH-dependent balance of FQ influx through porins or the lipid bilayer [72]. Specifically, Hfq has been shown to affect genes related to pH homeostasis, potentially influencing membrane transport mechanisms [67]. Positively charged ciprofloxacin can pass through the lipid bilayer without requiring porins [42,72]. Additionally, pH impacts the effectiveness of aminoglycosides, which is significantly diminished under low pH [73,74]. Indeed, pH is known to influence the electrical component of the proton-motive force (PMF), and increasing pH may potentially enhance the uptake of aminoglycosides. Note that during human tissue infections, bacteria must cope with an acidic environment, and Hfq may help them adapt to low pH. Finally, many antibiotics rely on some ions to be effective, a process regulated by Hfq [75]. For example, aminoglycosides require Mg^2+^ ions to bind to ribosomes and are less effective in low-magnesium environments [76]. A change in ion concentration may also alter the membrane potential and affect the functioning of antibiotic transporters [74]. Note that Hfq’s amyloid-like C-terminal region may alter the structure of Omps, potentially affecting the equilibrium between their β-barrel or amyloid forms by a cross-seeding mechanism [77,78,79].

Various studies have demonstrated more and more examples of the role of the Hfq-mediated regulation of gene expression, predominantly through either the stabilization or destabilization of certain RNA species, in the development of antibiotic resistance by different bacteria. An interesting example has been discovered in *E. coli*, where Hfq is required to enhance the stability of the RalA RNA, acting as an antitoxin to the RalR enzyme (a DNAase), which results in reduced sensitivity to fosfomycin [80]. A systematic analysis of the effects of as many as 26 small regulatory RNAs that require Hfq to decrease the susceptibility of *E. coli* to various antibiotics (gentamycin, carbapenem, cephalosporins, quinolones, and tetracycline) suggested that the main role of this protein in antibiotic resistance might be related to its RNA chaperone activity for a relatively restricted number of small RNAs [38,81]. This was identified as a promising starting point in searching for novel drug targets [82]. However, other discoveries have demonstrated that this path is not so simple. Namely, it was found that levels of ImpR, one of outer membrane proteins, are increased in *E. coli hfq* mutant cells, but such mutants revealed decreased susceptibility to carbapenem [83]; this was in contrast to previous reports indicating the increased, rather than decreased, sensitivity of Hfq-deficient cells to antibiotics (see above). Such an ostensible discrepancy might arise from the fact that Hfq is involved in the regulation of the expression of genes coding for both efflux pumps, like AcrAB [49], and porins, like the ChiP porin [84]. Moreover, Hfq was found to be able to modulate lipopolysaccharide (LPS) composition by interacting with RybB and MicA small RNAs [85]. In fact, LPS content varies under different environmental conditions that may significantly influence the susceptibility of bacteria to various agents. For example, in *Pasteurella multocida*, the presence of phosphoethanolamine in LPS is essential for resistance to cathelicidin-2, an antimicrobial peptide, while the expression of the *petL* gene, necessary for transferring phosphoethanolamine to lipid A, is negatively regulated by an Hfq-dependent small RNA [86].

Despite the above-mentioned decreased susceptibility of *E. coli hfq* mutants to carbapenem [83], other studies have reported that Hfq is required for the repression of the transcription of the *oprD* gene, coding for a porin in *P. aeruginosa*, which results in enhanced carbapenem resistance in the presence Zn^2+^ ions [87]. Again, one should take into account environmental conditions, exemplified here by levels of zinc, that can significantly modulate bacterial resistance to antibiotics. However, a subsequent report demonstrated an increased susceptibility of *P. aeruginosa hfq* mutants to different antibiotics, among others (like cephalosporins, aminoglycosides, fluoroquinolones, polymyxins, tetracyclines), carbapenems [88]. Another example of the Hfq-mediated stimulation of antibiotic resistance came from studies on *Neisseria gonorrhoeae*, where the overproduction of Hfq resulted in a lower susceptibility to ceftriaxone [89]. Decreased resistance to chloramphenicol was also confirmed in two separated studies after introducing an *hfq* mutation into *E. coli* [90], and in one of them, the absence of Hfq function was correlated to the regulation of *ompF* gene (coding for a porin) expression [91]. Levels of another *E. coli* porin, the production of which seems to be indirectly regulated by Hfq, the OmpC protein, correlated with susceptibility to antibiotics [92]. In *Shigella sonnei*, the small RNA-dependent regulation of the expression of the porin-encoding *ompD* gene, in which Hfq is required, was demonstrated to be linked to resistance to ampicillin, gentamicin and cefuroxime [93].

### 4.3. Involvement of Hfq in the Control of Bacterial Growth and Metabolism

When looking for general mechanisms of the Hfq-mediated enhancement of antibiotic resistance (or tolerance), it is worth noting that this phenomenon has been linked to the regulation of bacterial growth. In accordance with the studies demonstrating the enhanced resistance of bacteria by Hfq, it was shown that the deletion of the *hfq* gene in *Aeromonas veronii* resulted in retarded growth in cultures and higher susceptibility to several antibiotics [94]. In *P. aeruginosa*, Hfq influenced bacterial growth by limiting the toxic effects of the expression of genes regulated by the MexT transcription regulator [34]. Importantly, MexT also governs antibiotic resistance, making the role of Hfq in the resistance more complex than initially suspected. When the growth inhibition of *E. coli* and *K. pneumoniae* was induced by silencing some essential genes, also in the presence of carbapenem, the effects were more pronounced in *hfq* mutants [95].

Recent studies have pointed to the importance of the Hfq-dependent regulation of carbon catabolite repression in relation to antibiotic resistance in *P. aeruginosa* [96], though we are still far from obtaining a precise understanding of the molecular mechanisms of these interactions. Nevertheless, some insights were provided by discovering a specific role of the Crc protein. This protein stabilizes complexes between Hfq and its target RNAs. However, there is a small RNA molecule, called CrcZ, which is able to sequester the Crc-Hfq complex when there are no catabolic repression conditions. It was found that the deletion of the *crcZ* gene resulted in alterations in antibiotic resistance, while *crc* mutations could restore these changes [97]. Thus, the Hfq-interacting protein Crc may play a major role in Hfq-related linking metabolic regulations to antibiotic resistance, at least in *P. aeruginosa*.

### 4.4. Hfq-Dependent Control of Propagation of Mobile Genetic Elements Carrying Antibiotic Resistance Genes

In all of the above-described experimental systems, the studies focused on bacterial resistance systems based on general mechanisms, not specific to any antibiotics, like the formation of biofilm, regulation of efflux pumps and porins, or control of cell growth and metabolism. However, many antibiotic resistance properties of bacterial cells rely on activities of specific proteins causing insensitivity to certain antibiotics. These proteins are often encoded by genes located in mobile genetic elements, thus being prone to horizontal gene transfer and the spreading of the resistance feature. Can Hfq also be involved in the regulation of such kinds of antibiotic resistance?

Antibiotic resistance genes are often located in bacterial transposons. It was proposed that the Hfq protein may be involved in the negative regulation of the expression of genes coding for transposases (enzymes responsible for the translocations of transposons from one genome location to another) in transposons Tn5 and Tn10 [98]. Thus, Hfq might be involved in the control of the spreading of Tn5- and Tn10-borne antibiotic resistance genes.

Another type of vehicle capable of carrying antibiotic resistance genes is plasmids. The plasmid-located genes are responsible for resistance to relatively high concentrations of antibiotics as the products of these genes specifically inactivate antibiotics through their modifications or decay or by their active removal from the cell. In fact, resistance to especially high levels of antibiotics is important for bacteria when antibiotic therapy is conducted, particularly during the oral administration of the drugs when local concentrations of therapeutic compounds might be extremely high. Experiments conducted with *E. coli* strains harboring plasmids with genes responsible for resistance to different antibiotics demonstrated that Hfq enhances the survival of bacteria under conditions of very high concentrations of chloramphenicol, tetracycline, and ampicillin; this was concluded on the basis of the observation that wild-type bacteria were more resistant to these antibiotics than otherwise isogenic Δ*hfq* mutants [99]. Interestingly, Hfq-dependent resistance to high levels of tetracycline was related to the plasmid copy number, suggesting that Hfq may influence the levels of resistance by controlling plasmid DNA replication [99,100]. Indeed, the results of experiments with a Δ*rom* mutant of a ColE1-like plasmid corroborated such a possibility [99,101]. Another possibility is that Hfq modulates plasmid DNA replication by direct interactions with DNA, especially single-stranded fragments of this molecule. Indeed, such an activity of Hfq has been demonstrated experimentally [102].

Bacterial conjugation is one of the major ways of spreading plasmids and genes located in these mobile genetic elements. Recent investigations of a conjugative plasmid from the IncP-1α group identified a small regulatory RNA, named GadY, which regulates the plasmid-mediated conjugation when present in donor cells. Hfq binds GadY [103], strongly suggesting that this protein participates in the control of plasmid conjugal transfer, thus taking part in spreading antibiotic resistance genes present in conjugative plasmids.

### 4.5. Hfq in Gram-Positive Bacteria and Other RNA Chaperones in Light of Antibiotic Resistance

Finally, it is worth mentioning that *E. coli* Hfq homologs occur not only in Gram-negative bacteria but also in Gram-positive species, as summarized recently [104]. Such homologs can be exemplified by Hfq proteins identified in various bacterial species, including *Listeria monocytogenes* [105,106], *Staphylococcus aureus* [107,108], *Bacillus anthracis* [109,110], *Bacillus subtilis* [111], and *Clostridioides difficile* [112]. However, searching the PubMed database (see Section 2) for articles describing the influence of Hfq on antibiotic resistance in Gram-positive bacteria gave only one record, namely a paper presenting a discovery of Hfq-mediated, small RNA-dependent post-transcriptional regulations of gene expression processes in *Clostridioides difficile* [113]. This suggests that Hfq can also be involved in the control of resistance to antibiotics in Gram-positive bacteria. More studies in this field are definitely required. A recent discovery of the involvement of Hfq in oxidative stress response in *Listeria monocytogenes* [114] suggests that this protein can also play important roles under other stress conditions in Gram-positive bacteria, like under the plausible action of antibiotics.

Importantly, not only Hfq but also other RNA chaperones, like CsrA and ProQ, can participate in processes related to antibiotic resistance, as demonstrated recently in enteropathogenic *E. coli* [115]. Specifically, ProQ promotes resistance by stabilizing sRNAs that control stress response pathways and biofilm formation. On the other hand, CsrA influences antibiotic resistance by regulating genes involved in bacterial metabolism, efflux pump activity, and biofilm formation. Together, these factors help bacteria survive under antibiotic stress. Therefore, further studies on Hfq and related proteins appear to be promising in the course of searching for novel antibacterial therapies or those enhancing the efficacy of already known antibiotics. Indeed, methods allowing the determination of the effects and mechanisms of action of such proteins on antibiotic resistance are being optimized and developed [116].

## 5. Discussion and Conclusions

Studies from the last 20 years or so have indicated that the Hfq protein is involved in the regulation of antibiotic resistance in both Gram-negative and Gram-positive bacteria. The mechanisms of Hfq actions and effects on antibiotic resistance are summarized in Table 1. The molecular mechanism by which this protein controls the response of bacterial cells to the presence of antibiotics are complex and involve different functions of Hfq, including actions as an RNA chaperone, DNA binding, and interactions with membranes. The processes involved in the regulation of antibiotic resistance in which Hfq plays important roles are as different as biofilm formation, regulations of efflux pumps, porins, lipopolysaccharide composition, the control of cell growth and metabolism, and the modulation of propagation of transposons and plasmids. One should also take into consideration that the activity of Hfq can be significantly modulated by other factors; an interesting example is the recently discovered influence of the HqbA protein, which interferes with Hfq-sRNA interactions, especially when the binding of the Hfq protein to its target RNA is relatively weak [117]. Interestingly, HqbA is an acetyltransferase (modifying proteins rather than small molecules, thus perhaps unable to acetylate antibiotics), but its Hfq modulation activity is independent of enzymatic function [117], making the Hfq regulatory network even more complex. Nevertheless, it appears evident that Hfq is a major modulator of antibiotic resistance, and as such, it might be considered a potential target for novel antimicrobial drugs.

**Figure 2:**
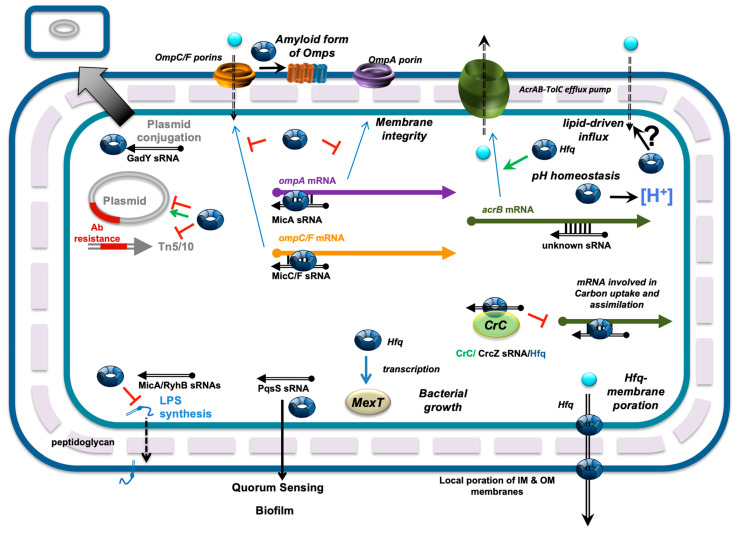
Network of Hfq-dependent regulations influencing antibiotic resistance. Hfq has been implicated in regulating the expression of genes involved in antibiotic resistance, primarily through the action of small noncoding RNAs (sRNAs). This applies to the regulation of efflux pumps, such as AcrB, which actively expel antibiotics from the cell. Hfq also influences antibiotic influx by regulating outer membrane proteins (Omps). For instance, the translation of *ompA*, *ompF*, and *ompC* mRNAs is directly repressed by the sRNAs MicA, MicF, and MicC, respectively. Hfq may additionally impact the amyloid-like conversion of Omps. In parallel, Hfq mediates biofilm formation, likely through the PqsS sRNA. Hfq also controls the composition of lipopolysaccharides (LPSs) by interacting with sRNAs. Furthermore, the regulation of bacterial growth through the modulation of the MexT transcription factor influences antibiotic resistance. Hfq is involved in the Hfq-Crc protein-CrcZ sRNA network, which regulates carbon catabolic repression, a process that also impacts antibiotic resistance. Hfq inhibits the expression of transposase genes in Tn5 and Tn10, thereby influencing the spread of Tn5- and Tn10-borne antibiotic resistance genes. It also regulates the replication of plasmids carrying antibiotic resistance genes and controls the conjugative transfer of plasmid DNA by interacting with the GadY sRNA. In addition to its role in sRNA-based regulation, Hfq directly affects fluoroquinolone accumulation, possibly by creating pores in the inner and outer membranes. Hfq may also influence fluoroquinolone influx indirectly by modulating pH homeostasis.

A network of Hfq-dependent regulations influencing antibiotic resistance is presented in Figure 2, providing a summary of our current knowledge on the role of this small protein in modulating the mechanisms that allow bacterial cells to survive in the presence of various antimicrobial agents. In brief, Hfq may influence antibiotic influx or efflux, membrane integrity, and pH homeostasis, as well as potentially changing lipid metabolism and membrane composition. Indeed, bacteria can resist antibiotics through several mechanisms, including the production of efflux pumps that actively expel antibiotics from the cell, the enzymatic degradation or modification of certain antibiotics (e.g., β-lactams), and reduced permeability by altering the cell wall or membrane structure. Since Hfq has been shown to play a role in all of these mechanisms, it could serve as a key target in the fight against multidrug-resistant (MDR) bacteria. This warrants further investigation to explore the potential of targeting Hfq N-terminus or C-terminus for the development of novel antibacterial drugs. By promoting the N-terminus function of Hfq while inhibiting its C-terminus self-assembly, which is responsible, for instance, for membrane pore formation, one could, for instance, enhance the efficacy of fluoroquinolones [41,42].

On the other hand, there are some potential difficulties in developing effective Hfq modulators that could work efficiently in increasing the susceptibility of bacteria to antibiotics. First, as indicated above, Hfq can affect both the influx and efflux of antibiotics; thus, a potential drug should be able to act only towards increasing the accumulation of antibacterial drugs in microbial cells, which perhaps will not be easy to achieve. Second, if one aims to develop a broad-spectrum Hfq-modulating antibacterial drug, it is necessary to consider differences between Hfq homologs in Gram-negative and Gram-positive bacteria [104]. Third, Hfq is a small protein (102 amino acid residues in *E. coli*), which could make finding specific small molecules interacting with this protein and changing its function(s) specifically quite challenging. Nevertheless, as discussed in this paper, Hfq is a promising target in searching for drugs that may enhance efficacy of antibiotics; thus, works on such potential therapeutics are highly encouraged.

While initial attempts typically focus on the Sm-fold region of the protein [28], alternative approaches could also be considered. Notably for Hfq proteins that harbors an amyloid region (such as those of *E. coli* or possibly those of *Acinetobacter baumannii* [118]), the repurposing of molecules developed to target amyloids in eukaryotic diseases presents a promising strategy to accelerate the discovery of a new class of antibiotics [119]. These compounds, which inhibit functional amyloid assemblies, could reduce preclinical and tolerance studies if they have been already conducted for amyloid-related human pathologies [120]. This approach has the potential to reduce both development costs and timelines for patient use [121]. By bypassing preclinical phases if the drug is already approved, the drug discovery process could be expedited [121,122]. As proof of concept, some amyloid inhibitors, including Epigallocatechin gallate (EGCG), apomorphine, and curlicide, have demonstrated antimicrobial properties, suggesting their potential utility in treating both amyloid-associated diseases and bacterial infections [30,123,124,125].

## Figures and Tables

**Table 1:** A summary of the roles of the Hfq protein in the regulation of bacterial resistance to antibiotics (the order of presentation reflects the alphabetical order of the names of bacterial species).

Species	Molecular Mechanism of Hfq Action	Effects of Hfq	References
*Aeromonas veronii*	Regulation of pathways including antioxidative defense (OxyR and RpoS), virulence (ClpB), toxin–antitoxin module (RelE and CspD), and transporter protein OppB by Hfq protein	Retardation of growth, reduction in tolerances to diverse antibiotics and persistence in *hfq* mutant	[94]
*Aeromonas veronii*	Regulation of AcrAB efflux pump at post-transcriptional level	Higher sensitivity to trimethoprim in *hfq* mutant relative to control variant Upregulation of purine metabolic genes, and downregulation of *acrA* and *acrB* genes in *hfq* mutant Complementation of *acrAB* genes causes results in recovery of trimethoprim resistance in Δ*hfq* cells More effective accumulation of adenosine and guanosine in bacterial cells with Hfq deficiency	[57]
*Clostridioides difficile*	Interaction and stabilization of Hfq proteins with sRNA molecules	Increased sensitivity to stresses, sporulation rates, and biofilm formation in Δ*hfq* mutant Utilization of ethanolamine is regulated by Hfq-dependent sRNA CDIF630nc_085	[113]
*Escherichia coli*	Mediation of RpoS-GadY(Hfq)-GadX signaling pathway in the activation of the *mdtEF* promoter	Lack of MdtEF-dependent drug tolerance of stationary-phase cells in *hfq* deletion mutant	[44]
*Escherichia coli*	Synergistic interaction of the Hfq protein with multiple signaling pathways and sigma factors, including RpoE and RpoS in uropathogenic *E. coli* bacteria (UPEC)	Reduction in motility, chemotaxis, and ability to form biofilms of *hfq* mutant Reduction in level of cell tolerance to RNS, ROS, polymyxin B, and acidic environment (pH 5)	[45]
*Escherichia coli*	Regulation of AcrAB efflux system at post-transcriptional level Positive regulation of production of AcrB protein without changes in promotor activity of *acrAB* operon	Increased drug sensitivity in *hfq* mutants (chloramphenicol, acriflavine, Crystal Violet, rhodamine 6G, benzalkonium, oxacillin, cefamandole or nalidixic acid) Impairment of effect of *hfq* deletion in *acrB* mutants	[49]
*Escherichia coli*	Positive regulation of RalA sRNA activity and its stabilization Interaction of RalA RNA (antitoxin) with mRNA of RalR (toxin—DNase) *via* base-pairing, thus preventing translation of RalR	Abolition of RalA activity in *hfq* mutant Increased sensitivity to fosfomycin in Δ*ralR* and Δ*ralRA* mutants	[80]
*Escherichia coli* and *Pseudomonas aeruginosa*	Negative regulation of *hfq* mRNA levels in both bacterial species by *Burkholderia cenocepacia* MtvR sRNA	Reduction in resistance to stress factors (SDS, NaCl, ethanol, and methyl viologen), and biofilm formation ability Increased susceptibility of cells to antibiotics (chloramphenicol, ciprofloxacin, tetracycline, tobramycin, gentamycin, and ampicillin)	[46]
*Escherichia coli* and other enteric bacteria	Downregulations of Tn5 transposase expression at transcriptional level by Hfq protein Inhibition of Tn10 transposase expression at post-transcriptional level by Hfq protein	Involvement of Hfq protein in control of Tn5- and Tn10-borne antibiotic resistance genes	[98]
*Escherichia coli* and *Salmonella* species	Interaction of Hfq protein with sRNA molecules in response to antibiotics	Abolition of resistant or sensitive phenotypes generated by overexpression of ChiX, CyaR, MicC, RybD, RyeB, and SgrS RNA molecules to all tested antibiotics in Δ*hfq* strains	[38]
*Escherichia coli*	Modulation of LPS composition by interactions of Hfq with RybB and MicA sRNA molecules in RybB-dependent manner	Structural alterations of LPS composition are critical for antibiotic resistance, OM integrity, virulence, survival in host, and adaptation to specific environmental niches	[85]
*Escherichia coli*	Interaction of Hfq with sRNA molecules	Disruption of Hfq-dependent small RNA regulation by mutations in the *hfq* and *chiX* genes, and overexpression of Chip porin Maintenance of resistance to different classes of antibiotics, including the carbapenems ertapenem and meropenem in mutants with high ChiP expression	[84]
*Escherichia coli*	Hfq-mediated control of plasmid replication Stabilization of sRNA transcripts by Hfq protein Interaction of Hfq with Rom (negative regulator of ColE1-like plasmid replication initiation) in regulation of replication of ColE1-like plasmid	More efficient replication of ColE1-like plasmids in *hfq* mutant at late exponential and early stationary phases Lack of effects of *hfq* deletion on ColE1-like plasmid replication in absence of *rom* gene	[100]
*Escherichia coli*	Regulation of multidrug efflux pump by Hfq-sRNA complexes	Tolerance to benzoate and sensitivity to chloramphenicol in *hfq* mutant	[91]
*Escherichia coli*	Indirect regulation of OmpC production by Hfq protein	Increased levels of OmpC in bacterial cells and susceptibility to antibiotics after Hfq knockout	[92]
*Escherichia coli*	Negative regulation of gene silencing effects in the sRNA system by Hfq protein	Inhibition of growth in *hfq* mutant	[95]
*Escherichia coli*	Regulation of expression of antibiotic resistance genes by Hfq protein Regulation of bacterial resistance to antibiotics by Hfq-dependent control of plasmid DNA replication Regulation of replication of ColE1-like plasmid by interplay between Hfq and Rom (negative regulator of ColE1-like plasmid replication initiation)	Higher survival rate of plasmid-containing bacterial cells against high concentrations of chloramphenicol (plasmid p27cmr), tetracycline (pSC101, pBR322) and ampicillin (pBR322) in presence of *hfq* gene Influence of plasmid copy number on Hfq-dependent bacterial cell resistance to high doses of tetracycline Lack of significant differences between efficiency of transformation of Δ*hfq::kan* and ΔCTR*hfq::kan* cells with pBR322, contrary to plasmid with *rom* deletion	[99]
*Escherichia coli*	Hfq binds to the ssDNA and influences recombination and replication processes	Promotion of formation of parallel helix by CTR Positive regulation of M13 replication and negative regulation of λ recombination in *hfq* mutans More pronounced of effect of Δ*CTR* mutation on both M13 replication and λ recombination in the case of Δ*hfq* Less efficient recombination of Δ*hfq* cells lacking both the NTR and CTR regions in contrast to only CTR-deficient cells	[102]
*Escherichia coli*	Promotion of RNA annealing and induction of a string alignment between RNAs and DNA in control of replication of ColE1-type plasmid by Hfq protein Destabilization of dsDNA duplex in two regions of ColE1 origin region by amyloid-like region (CTR) of Hfq protein Negative regulation of ColE1 plasmid replication by Hfq protein	Stabilization of double strand DNA ColE1 origin structure by CTR Aligning ColE1 origin dsDNA by CTR Acceleration of RNA I-RNA II annealing by CTR Aligning RNA I-RNA II duplex by CTR Stabilizing RNA I-RNA II duplex, especially at high temperatures, by CTR Stabilization of RNA II secondary structure but not that of RNA I by CTR CTR does not melt C-stretch region of ColE1 plasmid DNA Inhibition of plasmid replication initiation in *hfq* mutants	[101]
*Escherichia coli*	Inhibition of translation of *ompF* by MicF sRNA in Hfq-dependent manner	More effective accumulation of ciprofloxacin, irrespective of presence of functional AcrAB-TolC efflux pump in Δ*hfq* or Δ*ctr* mutants Impaired accumulation of ciprofloxacin in bacterial cells during overproduction of MicF sRNA which negatively regulates expression of *ompF* gene	[41]
*Escherichia coli*	Regulation of multidrug efflux pump by Hfq-sRNA complexes	Higher sensitivity to chloramphenicol (CHL) of original CHL-sensitive and CHL-resistant bacterial cells with deletion of *hfq* gene	[90]
*Escherichia coli*	Control of plasmid conjugal transfer by interaction of Hfq with GadY sRNA	GadY-dependent positive regulation of SM10lp-PAO1 conjugation by targeting the orphan LuxR-type receptor SdiA that decreases expressions of global repressors KorA and KorB in presence of quinolone antibiotics	[103]
*Escherichia coli*	Regulation of antibiotic transmembrane transport by Hfq protein independently of sRNAs molecules Regulation of membrane integrity and direct ciprofloxacin influx and/or pH homeostasis	Impaired accumulation of ciprofloxacin in *ompC* and *ompF* mutants in absence of Hfq or its C-terminal domain in bacterial cells	[42]
*Neisseria gonorrhoeae*	Regulation of efflux system at the post-transcriptional level	Lower susceptibility to ceftriaxone during overexpression of Hfq in bacterial cells	[89]
*Pasteurella multocida*	Negative regulation of *petL* expression by an Hfq-dependent sRNA molecule Involvement of Hfq and Fis in control of phosphoethanolamine (Petn) decoration of lipid A molecule	Increased expression level of *petL* gene in *hfq* mutant Regulation of bacterial cell resistance to antimicrobial peptide cathelicidin-2 by *petL and petK*, and the transfer of Petn on lipid A and Kdo1	[86]
*Proteus mirabilis*	Regulation of outer membrane protein, named ImpR by Hfq-MicM sRNA complex	Reduction in MicM RNA level in *hfq* mutant Increased level *of impR* mRNA in *hfq* and *micM* mutants Overexpression of ImpR resulted in higher resistance of bacterial cells to carbapenem treatment	[83]
*Pseudomonas aeruginosa*	Repression of transcription of *oprD* gene, coding for porin, in presence of Zn^2+^ ions Localization of CzcR to *oprD* promoter in Hfq-dependent manner Repression of *oprD* by Hfq, Cu and CopR (transcriptional regulator of CopRS two-component system)	Modulation of imipenem resistance as a consequence of metal response	[87]
*Pseudomonas aeruginosa*	Regulation of energy metabolism and c-di-GMP levels by Hfq Regulation of specific antibiotic resistance determinants *via* riboregulation or direct translational repression Regulation of several functions conferring susceptibility at the post-transcriptional level by the interaction of Hfq with Crc Sequestration of Hfq protein during overproduction or induction by non-preferred carbon source of CrcZ	Increased susceptibility of *hfq* deletion strains to different β-lactam (cephems/cephalosporins IV and penems/carbapenems), and non- β-lactam (aminoglycosides, fluoroquinolones, fosfomycins, lipopeptides/polymyxin, and tetracyclines) antibiotics	[88]
*Pseudomonas aeruginosa*	Hfq-dependent negative regulation of *hilR* gene expression (encodes small toxic protein) that is controlled by MetT transcription factor	Growth defect of *hfq* mutant cells	[34]
*Pseudomonas aeruginosa*	Stabilization of Hfq-mRNA complexes by Crc Modulation of activity of Hfq and Crc during catabolite repression by CrcZ	Abolition of catabolite repression phenotype by the mutation in *crc* gene Reduction in constitutive catabolite repression presented by the ∆c*rcZ* mutant by some mutations, likely decreasing affinity of Crc to Hfq/mRNA	[97]
*Pseudomonas aeruginosa*	Creation of Hfq/Crc/(target)RNA complexes and post-transcriptional regulation of carbon catabolite repression (CCR) Titration of Hfq and Crc by the CrcZ RNA and CrcA (catabolite repression control protein antagonist), respectively	Permission for translation of catabolic genes that were subject to translational repression by Hfq/Crc during CCR—metabolism of less preferred C-source(s) and continued growth	[96]
*Pseudomonas aeruginosa*	Formation of PqsS sRNA-Hfq complex that decreases *pqsL* mRNA stability by recruiting Rnase E to drive degradation.	Promotion of *pqs* quorum sensing (QS) Reduction in PAO1-mediated chronic infections including biofilm formation, antibiotic resistance, macrophage survival, and reactive oxygen species (ROS) production by PqsS sRNA	[32]
*Pseudomonas fluorescens*	Positive regulation of transcription of 2,4-DAPG biosynthetic gene *phlA* and AHL synthase gene *pcoI* by *hfq* gene Positive regulation of genes encoding flagellar biosynthesis factors, which are necessary for biofilm formation	Reduction in biofilm formation and colonization ability on wheat rhizospheres of the *hfq* mutant Reduction in level of 2,4-DAPG and AHL in *hfq* mutant	[47]
*Shigella sonnei*	Regulation of expression of genes belonging to two-component system, ABC transporters, ribosome, and biofilm formation Modulation of antibiotic susceptibility *via* multiple sRNAs Positive regulation of acid resistance genes: *gadA*, *gadB*, and *gadC* Positive regulation of *ipah9.8* gene from type III secretion system (T3SS)	Repression of most DEGs related to pathways of two-component system and biofilm formation in *hfq* mutant Increased bacterial cell sensitivity to antibiotics, including ribosome-targeting antibiotics, such as aminoglycosides and chloramphenicol in *hfq* mutant	[36]
*Shigella sonnei*	Stabilization of sRNA1039 and sRNA1600 by Hfq protein	Degradation of *cfa* mRNA in sRNA1039 deletion mutant and negative regulation of *ompD* gene (porin) expression Degradation of *tomB* mRNA in sRNA1600 deletion mutant and reduction in persister cells (lower resistance to cefuroxime), and decreased levels of genes expression from type III secretion system	[93]
*Yersinia pestis*	Regulation of the expression of many virulence- and stress-associated bacterial genes in cooperation with various sRNAs	Impaired ability of bacterial cells with *hfq* deletion to resist phagocytosis and survive within macrophages More effective attenuation of *hfq* mutant in mice subcutaneous or intravenous injection Positive regulation of Hfq protein on bacterial growth in response to heat, oxidative stress, nutrition limitation and antibacterial peptide (polymyxin B) Significant alteration in 243 genes in *hfq* mutant, about 23% of which are related to bacterial stress resistance and virulence	[48]

## Data Availability

This review article contains no newly created data.

## Abbreviations

The following abbreviations are used in this manuscript: Omp: outer membrane protein; sRNA: small noncoding RNA; IM/OM: inner/outer membrane; PG: peptidoglycan; OMV: outer membrane vesicle; Tn: transposon; FQ: fluoroquinolone; PMF: proton-motive force; PI: propidium iodide; LPS: LipoPolySaccharide; MDR: multidrug-resistant.
